# Are People-Centered Intelligences Psychometrically Distinct from Thing-Centered Intelligences? A Meta-Analysis

**DOI:** 10.3390/jintelligence9040048

**Published:** 2021-09-30

**Authors:** Victoria M. Bryan, John D. Mayer

**Affiliations:** Department of Psychology, University of New Hampshire, McConnell Hall, 15 Academic Way, Durham, NH 03824, USA; Jack.Mayer@unh.edu

**Keywords:** people-centered intelligences, broad intelligences, meta-analysis, socio-emotional abilities

## Abstract

The Cattell–Horn–Carroll (CHC) or three-stratum model of intelligence envisions human intelligence as a hierarchy. General intelligence (*g*) is situated at the top, under which are a group of broad intelligences such as verbal, visuospatial processing, and quantitative knowledge that pertain to more specific areas of reasoning. Some broad intelligences are people-centered, including personal, emotional, and social intelligences; others concern reasoning about things more generally, such as visuospatial and quantitative knowledge. In the present research, we conducted a meta-analysis of 87 studies, including 2322 effect sizes, to examine the average correlation between people-to-people intelligences relative to the average correlation between people-to-thing-centered intelligences (and similar comparisons). Results clearly support the psychometric distinction between people-centered and thing-centered mental abilities. Coupled with evidence for incremental predictions from people-centered intelligences, our findings provide a secure foundation for continued research focused on people-centered mental abilities.

Intelligence researchers of the 20th century debated whether intelligence was best conceptualized as a general reasoning capacity, first proposed by Charles Spearman (1904), or a set of more-or-less distinct mental abilities that ranged from verbal skills to spatial reasoning, as suggested by L. L. Thurstone (1938). The controversy centered on the empirical discovery that a *positive manifold* characterized the correlations among people’s ability to solve problems across diverse areas. That is, people’s ability to solve distinct types of problems rose and fell together: if a person were high on one mental ability, they tended to be high on them all.

Psychologists of the time reasoned that if the correlations among verbal, visuospatial, and other intelligences were near *r* = 1.0, all mental abilities were in perfect synchrony or nearly so across people. Such a state of affairs would argue decisively for Spearman’s general intelligence. At the other extreme, had the correlations been near *r* = 0.00, each mental ability would have been unambiguously distinct from the others, arguing for a theory of independent, multiple intelligences. As it turned out, however, the average correlations were closer to *r* = 0.30 to 0.50, leading to a degree of uncertainty as to whether intelligence was unitary or multifaceted. Spearman and his many followers (Gottfredson 1997; Jensen 1998; Ree and Carretta 2002; Spearman 1904) argued eloquently for a general intelligence. However, Thurstone, Guilford, and others countered with equally compelling evidence for the existence of distinct mental abilities (Guilford 1966; Thurstone 1938).

By the 1970s, the development of confirmatory factor analysis allowed for the modeling of hierarchical relations among mental abilities (e.g., Hu and Bentler 1999; Joreskog 1969); these techniques allowed for a more nuanced understanding of the positive manifold: that there existed both a general intelligence *and* distinct, broad intelligences that were worthy of study. The apotheosis of this new look was the *Cattell–Horn–Carroll* (CHC), or *three-stratum*, model of intelligence (Carroll 1993; Flanagan and Dixon 2014; McGrew 2009). The three strata refer to the fact that *g,* general intelligence, is enshrined at the top of a hierarchy—a chief executive officer, of sorts—under which are situated a set of 10 to 15 broad intelligences. Each broad intelligence, in turn, is measured by specific tasks, represented at the lowest, third tier of the model.

A number of these second-stratum broad intelligences concern reasoning with particular classes of symbols such as words or spatial images. For example, comprehension knowledge divides into vocabulary knowledge, sentence comprehension, and word fluency at the third stratum (Schipolowski et al. 2014); visuo-spatial ability divides into paper-folding and mental rotation; quantitative intelligence into tests of arithmetic. Also present in the second stratum are more foundational, process-based intelligences such as short-term memory, long-term retrieval, and mental speededness that reflect more general characteristics of reasoning (Kovacs and Conway 2016; Schneider and McGrew 2018). 

## 1. Organizing the Broad Intelligences 

Although the CHC model produced a viable compromise between advocates for *g* and for multiple intelligences, there was a fly in the ointment. As the number of identified broad intelligences proliferated from 8 to 12 or more, some researchers asked whether there were “too many intelligences” (Austin and Saklofske 2005; Hedlund and Sternberg 2000). To address this issue, psychologists have suggested that there may be subsidiary groups among the broad abilities that can help organize them. 

Perhaps the most well-known division among broad intelligences is that between fluid and crystallized intelligence (Cattell 1943; Cattell and Horn 1978; Horn and Cattell 1966, 1967). Fluid intelligence describes a general capacity to understand abstract relationships such as similarities and differences, apart from prior learning. Crystallized ability, by comparison, describes the depth, breadth, and understanding of acquired information about the world (Cattell 1961). These two factors are often found when analyzing broad intelligences, and are a precursor of the current CHC model (Carroll 1993). More recent proposals to organize the broad intelligences exist as well: Schneider and Newman (2015), for example, distinguished *power* intelligences including acquired knowledge and other domain-specific areas of reasoning from more *speeded* intelligences, reflecting the rate at which one finds an answer to a problem (Schneider and McGrew 2018; see also Figure 4 in Schneider and Newman 2015). A quite different model, developed by Mayer and colleagues, suggested the potential existence of a *people-versus-thing* continuum of broad intelligences—which will be our focus here (Mayer 2018; Mayer et al. 2016; Mayer and Skimmyhorn 2017).

### The People versus Thing Continuum

**Figure 1:**
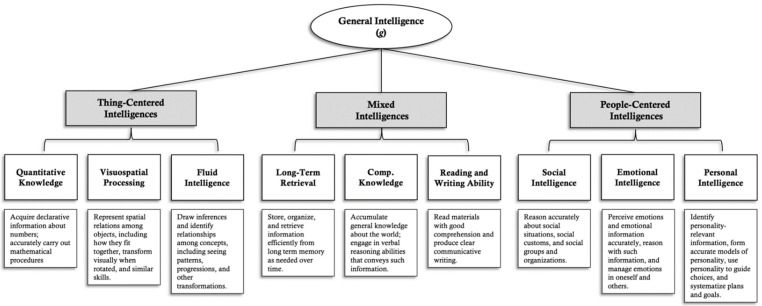
Modified CHC model organizing the broad intelligences across a people–thing continuum. In the model, a subset of broad intelligences are arranged along a continuum from topics of reasoning that primarily concern things to those that concern people.

Mayer and colleagues’ conception of the people-versus-thing continuum begins by noting that “many of the broad intelligences relate to specific subject or topic areas” such as quantitative, visuospatial, and verbal-comprehension areas (Mayer 2018, p. 272). There also exist content-free, process-based intelligences such as working memory and speededness, which lay outside the continuum but are important basic processing abilities (or “utility intelligences”) that people draw on in some capacity to solve many types of problems (Kovacs and Conway 2016). The people–thing continuum is focused on the content-focused broad intelligences and separates out people-centered problem-solving abilities like emotional, social, and personal intelligence—that individuals use to reason about themselves and others—from the other more thing-centered intelligences (Bryan and Mayer 2017; Mayer 2018; Mayer and Skimmyhorn 2017). The thing-centered group includes most centrally quantitative knowledge that concerns understanding numbers, and visuospatial processing, which pertains to understanding visual patterns as well as the movement of objects in space. In between the people- and thing-centered intelligences are mixed intelligences such as verbal–comprehension and reading–writing, which concern both people and things. This people–thing continuum is represented visually in the context of the modified Cattell–Horn–Carroll (CHC) model depicted in Figure 1.

## 2. Are People- and Thing-Centered Intelligences Truly Distinct?

The controversy addressed in this paper concerns whether the proposed people-centered abilities such as social, emotional, and personal intelligences are distinct in any fashion from other broad intelligences or from *g*. For example, Lee J. Cronbach wrote in the 1960s of social intelligence that “enough attempts were made [to measure it] to indicate that this line of approach is fruitless” (Cronbach 1960, p. 319). Indeed, the inability of many early researchers to produce evidence that social intelligence was psychometrically distinct from “abstract”, i.e., general intelligence (see R. L. Thorndike and Stein 1937) served to tamp down research in the area for decades (Conzelmann et al. 2013; Walker and Foley 1973). Emotional intelligence, too, had its early critics (Davies et al. 1998; Ree and Carretta 2002; Schulte et al. 2004), although it is now widely accepted as a broad intelligence within the field of intelligence (MacCann et al. 2014). Personal intelligence appears promising as a further semi-independent people-centered intelligence. However, is such optimism about the distinctness of this group warranted?

### 2.1. An Understanding of People-Centered Intelligences Is Just Now Emerging

Whether a people–thing continuum exists is still unexplored primarily because the class of people-centered intelligences has only recently become defined. An informal timeline of people-centered intelligences (which foregrounds brevity relative to subtlety) begins with the introduction of social intelligence in 1920 (E. L. Thorndike 1920), proceeds to its mid-20th-century demise as an area of interest (Cronbach 1960; Walker and Foley 1973), and picks up again with studies of nonverbal communication of emotion in the 1980s (Buck 1984). Our timeline marks the rise of emotional intelligence in 1990 (Mayer et al. 1990; Salovey and Mayer 1990), and the early controversy over whether it could be viably measured (Davies et al. 1998; Mayer et al. 1999; Zeidner et al. 2001)—finally settled in its favor. Still, 18 years after that, personal intelligence was introduced in the late 2000s, with the first measure in 2012 (Mayer 2008; Mayer et al. 2012). Concurrently, researchers increasingly regarded nonverbal emotion perception, now called emotion recognition ability (ERAs), as an intelligence itself, perhaps part of emotional intelligence (Schlegel et al. 2019b). The need for a new class of these measures was more recent still, coalescing in the past few years. 

The identification of people-centered mental abilities may be ongoing: certain cognitive and social-cognitive tasks have not yet been conceptualized as intelligences but perhaps ought to be further explored (Haier 2017, pp. 124–25). These might include wisdom and even spiritual intelligence; that said, they are not yet ready for inclusion in this review because there are few or no ability-based measures of these skills that have been related to intelligence.

Given the relative recency of the study of people-centered intelligences, the correlations among them reported in the research literature were relatively sparse until recently. There are now, however, enough such reports to allow for a meta-analysis that might answer at least one fundamental and crucial question: do people-centered intelligences correlate among themselves more highly than with thing-centered intelligences, and, similarly, do thing-centered intelligences correlate among themselves more highly than with people-centered intelligences? Put another way, are these two classes of intelligences partially distinct from one another or, as was apparently the case for social intelligence decades ago, are they indistinguishable from any other broad intelligence? 

### 2.2. Evidence for Incremental Validity Is Strong, but Also Incomplete and Indirect 

It is worth noting before proceeding that one set of findings already supports the possible existence of a people–thing continuum among intelligences: a growing body of research indicates people-centered intelligences incrementally predict selected criteria over and above thing-centered abilities. For example, personal intelligence predicts such criteria as positive interpersonal relations, better performance in people-centered college courses—but not STEM courses—and other theoretically identified relations over and above verbal, quantitative, and visuospatial abilities (e.g., Bryan 2018; Mayer et al. 2018; Mayer and Skimmyhorn 2017). Similar incremental evidence can be found for emotional intelligence (Mayer et al. 2008). Yet, although these findings argue for the importance of people-centered reasoning, such incremental validity could emerge owing to artifacts such as additional reliable variance over the original measure (e.g., Hunsley and Meyer 2003; Sechrest 1963; Westfall and Yarkoni 2016). The current meta-analysis will provide more direct evidence for or against the class of people-centered intelligences. 

## 3. Current Research

We set out to discover whether, within the pattern of positive correlations among broad mental abilities, there exist variations in correlational level supportive of the people–thing continuum. More specifically, we tested whether people-centered intelligences correlate more highly among themselves than with thing-centered intelligences, and whether their correlation with mixed intelligences lies between the two. We tested the reverse as well: that thing-centered intelligences correlate more highly among themselves than with mixed or with people-centered intelligences. 

To accomplish this, we reviewed the literature reporting correlations between people-centered ability-based intelligence measures with other mental abilities. Our meta-analytic work draws on and extends previous research in the area by including social and personal intelligences along with the more studied emotional intelligence (Olderbak et al. 2018; Schlegel et al. 2019b). The inclusion of a wider scope of people-centered intelligences has the benefit of allowing us to model the variability in relations among different types of people-focused reasoning in addition to understanding their relations as a group with other mental abilities. 

## 4. Hypotheses

With this in mind, we tested the following hypotheses:

**Hypothesis** **1** **(H1).** *People-centered intelligences will correlate most highly among themselves, next most highly with mixed intelligences, and least highly with thing-centered intelligences.*

To test this hypothesis, we examined the differences between the average correlations for people-to-people, people-to-mixed, and people-to-thing mental abilities.

**Hypothesis** **2** **(H2).** *Thing-centered intelligences will correlate most highly among themselves, next most highly with mixed intelligences, and least highly with people-centered intelligences.*

Hypothesis 2 is the complement of Hypothesis 1. We examined the differences between the average correlations for thing-to-thing, thing-to-mixed, and people-to-thing intelligences.

**Hypothesis** **3** **(H3).** *Personal and emotional intelligences will exhibit a greater difference (i.e., lower correlation) with thing-centered intelligences than social intelligence.*

In the past, researchers have had particular difficulty distinguishing social intelligence from general intelligence (see the Introduction section). Consistent with those findings, we expected that social intelligence would correlate more highly with thing-centered intelligences than either emotional or personal intelligence. We tested this by comparing the average correlations of measures of emotional, personal, and social intelligences with thing-centered intelligences, expecting the social-to-thing correlation to be highest. 

## 5. Additional Analyses

It was possible to construct a very limited correlation table among thing- and people-centered broad intelligences from our data; although the table’s utility is arguably limited by the fact that each correlation was drawn from different numbers and types of studies, we explored the possibility of factor analyzing the table to see if a people–thing factor emerged. Finally, we checked for publication bias among our sample of studies. 

## 6. Method

### Pre-Literature Search Index of People-Centered Assessments

Prior to beginning our literature search, we developed an index of ability-based assessments for emotional, social, and personal intelligences by noting, first, well-known tests in each area. For example, the *Mayer–Salovey–Caruso Emotional Intelligence Test* (MSCEIT) and *Situational Test of Emotional Understanding and Emotional Management* (STEU and STEM) are well-established ability-based assessments of emotional intelligence, the *George Washington Social Intelligence Test* is a known measure of social intelligence, and the *Test of Personal Intelligence* (TOPI) is an ability-based measure of personal intelligence (Allen et al. 2015; Mayer et al. 2003, 2019; Walker and Foley 1973). 

Our review omits potential measures of personal intelligence such as person memory, scales of wisdom, spiritual intelligence, interpersonal judgment accuracy, and empathic accuracy (e.g., Ickes 2016; Letzring and Funder 2018). Either there existed no ability-based measures in the area, or, as far as we knew, no report existed that correlated such measures with another ability-based intelligence measure.

We next consulted relevant review articles, book chapters, and meta-analyses for additional assessments. At a most fundamental level, any scale included in our index needed to be plainly operationalized as an ability-based test, i.e., with correct and incorrect answers. This ruled out self-judgment measures of intelligences such as the Tett, Schutte, and Bar-On scales of emotional intelligence (Bar-On 1997; Schutte et al. 1998; Tett et al. 2005), as well as the Self-Estimated Personal Intelligence measure (Mayer et al. 2021). At least one test fell in a gray area: the *Levels of Emotional Awareness Scale* (Lane et al. 1990). An earlier review remarked it “does not fit easily into the self-report personality category, the ability category, or the self-reported ability category” (Ciarrochi et al. 2003, p. 1488). Rather it may be closer to a cognitive style or thematic/projective measure and was omitted as a consequence. 

Finally, to be indexed, the scale had to possess a reasonable track record including one or more reports of reliabilities and correlations with other scales of intelligence; this tended to exclude both rarely used scales and new scales for which a research track record had not yet accumulated. 

Using the above procedures, we identified additional ability-based assessments for emotional intelligence from Rivers et al. (2007), Schlegel et al. (2019b), and Olderbak et al. (2018). Further measures of social intelligence were gathered from the review by Walker and Foley (1973), and more recent assessments in Conzelmann et al. (2013). A full list of indexed measures can be found in Table 1. 

## 7. Literature Search 

Drawing on the people-centered assessments in Table 1, we conducted a series of keyword searches using the full name of each ability-based assessment of emotional, social, and personal intelligence in Table 1, and entered it into PsycINFO to identify relevant works that correlated the measures of people-centered intelligences with one or more mixed or thing-centered assessments. This yielded over 4000 potentially relevant works. We also included an additional 167 articles we identified from three earlier meta-analyses on related issues (Bryan and Mayer 2020; Olderbak et al. 2018; Schlegel et al. 2019b). 

## 8. Inclusion Criteria

**Figure 2:**
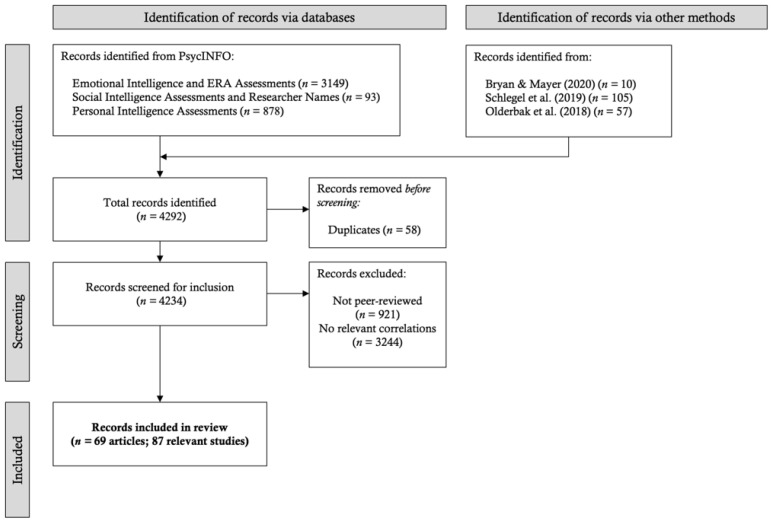
Article identification and screening process.

For each set of search results, the first author read through the titles and abstracts, and excluded irrelevant and/or duplicate articles that had emerged from previous searches. Each article was then screened further, on the basis of a series of inclusion criteria (see middle Figure 2). For inclusion, the work had to (a) be a peer-reviewed journal article, (b) employ at least one ability-based assessment of people-centered ability (i.e., emotional, personal, or social), and (c) report at least one Pearson correlation across possible types of comparisons (i.e., people-to-people, people-to-mixed, or people-to-thing). Although some experts have suggested that beta coefficients can be used to impute correlations in meta-analysis (e.g., Peterson and Brown 2005), more recent evidence argues against their use (Roth et al. 2018), as well as against the use of partial correlations more generally (Aloe 2014). Therefore, our focus was on identifying only zero-order Pearson correlations.

Sixty-nine articles reporting 87 studies met these criteria. The list of included studies can be found in Table 2.

## 9. Coding of Articles

The first author coded all 87 studies, with the assistance of a trained undergraduate research assistant who coded approximately half of the studies. The studies coded by both the first author and the research assistant were cross-checked to ensure coding accuracy. Discrepancies were resolved through discussions between the author and research assistant or by consulting the respective studies. Each study was coded for (a) year of publication; (b) sample characteristics (including sample type, average age, and gender); (c) the specific measure(s) used for each type of intelligence; (d) the reliabilities of each measure, if provided; (e) the correlation reported between people, mixed, or thing-centered intelligences; and (f) the sample size associated with each correlation. 

Additionally, while the people–thing continuum sets aside more foundational intelligences such as working memory and processing speed, we noted several assessments in our sample of studies that reported correlations with such abilities. In instances such as this, we recorded the measures used to assess both abilities as well as any relevant correlations with people-, mixed, or thing-centered abilities. Any correlations with working memory and processing speed were kept separate from any people-to-thing analyses discussed here, although we include them in separate analyses presented later (see More Specific Comparisons in the Results). See also Bryan and Mayer (2021b) for the full open-source data set and (Bryan and Mayer 2021c) for the full R script. 

### 9.1. Designation of Assessments as People-Centered, Mixed, or Thing-Centered

People-centered assessments included any measures of social, emotional, or personal intelligence as earlier indexed in Table 1. We then drew on theoretical work by Mayer (2018) and the subgroups of intelligences noted by Bryan and Mayer (2020) to inform our designation of assessments as people-, mixed, or thing-centered. A measure was designated as “mixed” if it assessed skills underlying either comprehension knowledge, reading and writing ability, or long-term retrieval, because these skills pertain to both people and things. Thing-centered assessment included visuospatial processing, quantitative knowledge, and measures of fluid intelligence because those often involve deciphering abstract patterns (e.g., Raven’s Matrices; Raven 2009).

When the immediate classification of a scale was uncertain, the two authors discussed the instance, proceeding through such matters on a case-by-case basis. For example, the Reading the Mind in the Eyes scale, for which respondents examine a rectangular area around the eyes of faces and then describe what is conveyed by it, includes response alternatives involving emotion recognition (e.g., irritated), but also items pertaining to emotion-related traits (comforting) and motivational or behavioral-descriptive traits (e.g., playful). In this instance, we classified the test as a measure of emotional intelligence because a number of items pertain to emotion recognition and the scale is frequently used as an ERA; however, there was arguably justification to place it with personal intelligence. Each assessment and its respective designation as people, mixed, or thing can be found in the “Full List of Included Works” section of the Technical Supplement (Bryan and Mayer 2021a). See also the “Designation of Assessments” in the Technical Supplement for additional information regarding the designations of more ambiguous assessments.

### 9.2. Distinguishing between Broad and Specific Assessments of Abilities

Each study was reviewed further, and correlations were distinguished according to whether they were between individual tasks, or between broad assessments, or between an individual task and a broad assessment. For example, studies that employed the Mayer–Salovey–Caruso Emotional Intelligence Test (MSCEIT) could have used the total EI score (a broad-based score), one or more of the eight individual task scores (e.g., Blends; Mayer et al. 2003), or something in-between (i.e., branch scores). Akin to the broad and narrow abilities reflected in the Cattell–Horn–Carroll (CHC) model, broader assessments included measures that tapped into multiple areas of reasoning within a broad ability (e.g., MSCEIT total scores are calculated from participants’ performance on all tasks involved in all four areas of emotion reasoning) while more narrow assessments were those that tapped into a single area of problem solving (e.g., emotion recognition; see Appendix A for our designations of assessments).

Correlations for the broader measures of a mental ability were common across assessments of people-centered abilities (e.g., MEIS total, GWSIT total, GECo total, etc.), but less so for the measures pertaining to mixed and thing-centered areas of reasoning, which tended to focus on more narrow skills (e.g., Raven’s Matrices, Wordsumplus). We matched scale types for each article where possible, for example, pairing a MSCEIT branch score, if reported, to Raven’s Matrices, as opposed to employing the more heterogeneous overall MSCEIT score. The only instance where we did not match scale types was when a study included only a correlation between mismatched measures (e.g., broad and narrow). Fortuitously, for the studies to date, the process outlined above left no window for selecting between two correlations that equally met criteria but differed in magnitude. For that reason, this process was independent of any potential researcher bias.

To ensure this process did not impact our results, we ran a separate set of analyses, including any broad assessments not included due to our method of matching of scale types. The findings were not substantively different from those reported here. See the “Distinguishing Between Broad and Specific Assessments” section of the Technical Supplement (Bryan and Mayer 2021a). 

### 9.3. Coding Intelligence Contrasts

Each correlation between a pair of intelligence scores represented one of six comparison types: a people-to-people correlation, people-to-mixed, people-to-thing, thing-to-thing, thing-to-mixed, or mixed-to-mixed correlation. We dummy coded those comparisons using six variables that could take on a 0 or 1 depending upon which of the comparisons was present.

This process led to the identification of 1973 effect sizes reflecting correlations between people-to-people, people-to-mixed, and people-to-thing intelligences. Three-hundred and forty-nine additional correlations were found between mixed-to-mixed, mixed-to-thing, and thing-to-thing intelligences, for a total of 2322 effect sizes.

## 10. Statistical Analyses

All effect sizes and standard errors were corrected for attenuation due to unreliability (Schmidt and Hunter 2015; Wiernik and Dahlke 2020). Reliability estimates used to disattenuate effect sizes and standard errors were predominantly obtained from the studies in which a given effect size was reported. However, in instances where the reliability of a measure was not reported, a value was estimated by drawing on either the measure’s reliability as indicated in other studies included in our sample, or by consulting other sources (e.g., test manuals, journal articles). Effect sizes were then transformed to Fisher’s Z’s and entered into the meta-analytic software to carry out tests of the central hypotheses.

We conducted a three-level multilevel meta-analysis using the *metafor* package in R (Viechtbauer 2010), where the Fisher’s Z-transformed, disattenuated effect sizes were nested within studies to account for any statistical dependence due to the nesting of multiple effect sizes within studies (Konstantopoulos 2011). Each analysis involved entering the relevant dummy-coded predictors reflecting different intelligence correlation contrasts (e.g., people-to-thing or thing-to-thing correlations) as moderators into the model (Enders 2013). Similarly, we created dummy codes for more specific comparisons (e.g., emotional intelligence-to-fluid intelligence). Following recommendations by Hall and Rosenthal (2018), all estimates of the average correlation among different intelligence contrasts were taken from the unweighted random effects model, which produces average effect estimates that are more generalizable across research methods. All reports of average estimated correlations between the different intelligence pairs have been transformed backed to disattenuated Pearson *r*s in this article.

## 11. Results

### 11.1. Study Characteristics

The 87 studies included in our analyses spanned from those published in the 1930s that examined social intelligence to studies published in 2020 examining emotional intelligence. A total of 50 of the 87 studies included in our sample (57%) were published during or after 2010, reflecting the recent upsurge of research in people-centered intelligences. Across the studies, sample sizes ranged from as few as 24 participants to more than 4000, for an overall sample size of 24,627 (*M =* 283.07). Samples were predominantly comprised of college students (58 studies) but with some child/adolescent, community, and other samples. The overall average age of participants was 25.52 years. Of the 83 studies that reported information regarding gender, samples were on average 56% female (Nmale = 9773, Nfemale = 12,439). Scale reliabilities for social intelligence assessments averaged α = 0.63 (range = 0.10 to 0.98), for emotional intelligence, α = 0.74 (range = 0.42 to 0.95), and for personal intelligence, α = 0.87 (range = 0.71 to 0.94; see Table 3).

### 11.2. Types of Abilities Represented

The number of effect sizes reflecting each type of people-centered intelligence followed their order of introduction in the field of intelligence, with social intelligence first at 1108, closely followed by emotional intelligence at 1053, and most recently by personal intelligence at 14 effect sizes. The people-centered intelligences were compared 424 times with mixed ability measures such as WAIS Vocabulary and SAT Verbal, and 464 times to thing-centered abilities such as Raven’s matrices, SAT-Math, and O*Net spatial.

## 12. Preliminary Analyses

### 12.1. Examination of Outliers

Prior to entering the effect sizes into our model, we examined the disattenuated-for-reliability (but not yet Fisher’s Z-transformed) correlations for the presence of outliers, defined as +/− 3 standard deviations from the mean. Four effect sizes, all from the same study, were flagged but their removal did not substantively affect the pooled correlation estimates or the confidence intervals, and the data were therefore retained.

### 12.2. Examination of between (Level 3) and Within-Study (Level 2) Heterogeneity

We drew on the intercept-only model to calculate heterogeneity estimates for the distribution of variance across levels of our model. The majority of heterogeneity was attributed to within-study (level 2; *I*^2^ = 65.05%) variance, followed by between-study (level 3; *I*^2^ = 30.81%) variance. Heterogeneity estimates were significant at both level 2 and 3 (*p’s* < 0.001). The effect sizes varied sufficiently within and between studies as to imply the presence of moderators (Assink and Wibbelink 2016). For this reason, we proceeded to test our hypotheses.

## 13. Test of Hypotheses

### 13.1. People-Centered Intelligences Will Correlate Most Highly among Themselves, Next-Most-Highly with Mixed Intelligences, and Least Highly with Thing-Centered Intelligence (Hypothesis 1)

**Figure 3:**
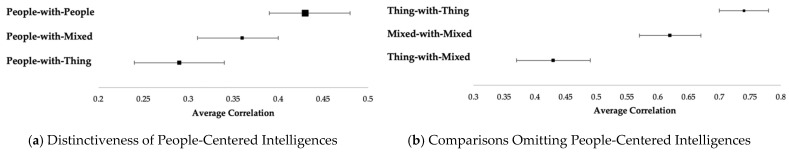
A Comparison of the Distinctiveness and Relatedness of the Six Types of Broad Intelligences Examined Here. Estimates relative to people-centered intelligences are to the left (**a**); those for the remaining categories are to the right (**b**). The average correlation estimates across studies were taken from the random effects model. The box sizes of the forest plots reflect the relative number of effect sizes (k) associated with a given estimate. The specific numerical values of the effect sizes, sample sizes and point estimates for each contrast can be found in Table 4.

To test whether people-to-people measures were more highly correlated relative to people-to-mixed and people-to-thing comparisons, we entered the three dummy-coded comparison types as moderators into our model and found significant differences between the pairings (*F*(3, 2318) = 78.78, *p <* 0.001) (see Figure 3a). As predicted, people-centered intelligences exhibited average correlations more highly among themselves, at *r* = 0.43, 95% CI [0.39, 0.48], compared to *r =* 0.36, 95% CI [0.31, 0.40] for people-with-mixed, and *r* = 0.29, 95% CI [0.24, 0.34] for people-with-thing correlations. Recall that these correlations are corrected for attenuation due to reliability; all the differences among the comparisons were significant at the *p* < 0.001 level or beyond. Sample and effect size information for each of the people-centered intelligence contrasts can be found in Table 4. 

### 13.2. Thing-Centered Intelligences Will Correlate Most Highly among Themselves, Next-Most-Highly with Mixed Intelligences, and Least Highly with People-Centered Intelligences (Hypothesis 2)

Did Hypothesis 2 hold also? That is, did thing-centered intelligences demonstrate similarly high within-group relations relative to the others? We carried out a second analysis in which the dummy-coded contrasts reflecting thing-to-thing, thing-to-mixed, and people-to-thing comparisons were then entered as moderators in our model. The three yielded significant between group differences overall (*F*(3, 2318) = 93.55, *p* < 0.001). As predicted, thing-centered intelligences correlated most highly among themselves (*r* = 0.74, 95% CI [0.70, 0.78]), followed next by mixed intelligences (*r* = 0.43, 95% CI [0.37, 0.49]), and least with people-centered intelligences (*r* = 0.29, CI [0.24, 0.34]; see Figure 3b). Pairwise comparisons revealed that the average correlation among thing-centered abilities was significantly higher than the average correlations among mixed-to-thing and people-to-thing intelligences (all *p’s* < 0.001). Sample and effect size information for each of the thing-centered and mixed intelligence contrasts can be found in Table 4.

#### 13.2.1. More Specific Comparisons

As noted in the earlier “Statistical Analysis” section, we had further coded for more specific groups of intelligences. Table 5 shows the average correlation of social, emotional, and personal intelligences (top row) with more specific groups of intelligences. For example, mixed intelligences are divided into comprehension knowledge, long-term memory, and reading and writing types. Additional correlation estimates comparing general assessments of emotional intelligence and more specific emotion recognition ability (ERA) assessments can be found in the “Breakdown of Emotional Intelligence Measures” section of the Technical Supplement (Bryan and Mayer 2021a). That breakdown indicates that general emotional intelligence and ERAs exhibit similar patterns to one another and to other intelligences. Yet although measures of general emotional intelligence formed a relatively cohesive group, correlating with one another at *r* = 0.52, as did ERAs among themselves at *r* = 0.57, the two sets of measures were a bit less related when compared one to the another (*r* = 0.33).

#### 13.2.2. Average Correlations among People-Centered Intelligences 

The intercorrelations among people-centered abilities varied (*F*(4, 2317) = 16.66, *p* < 0.001) (see top rows, Table 4): Emotional intelligence correlated most highly with personal intelligence (*r* = 0.70) and least with social intelligence (*r* = 0.23), a statistically significant difference (*p <* 0.010). The within-group average for emotional intelligence (*r* = 0.50) also was significantly higher than the social intelligence assessments (*r* = 0.33; *p* < 0.001). No data were present regarding the correlation between personal and social intelligences.

#### 13.2.3. Average Correlations among People-Centered and Mixed Intelligences 

The average correlations for social, emotional, and personal intelligences with assessments of comprehension knowledge were also robust and comparable across each person-centered ability (range = 0.35 to 0.41, *p’s*, n.s.). Some variability did exist in the correlations between each people-centered ability with reading and writing ability, most likely due to the number of available effect sizes for each contrast (see middle rows, Table 5). Indeed, while the average estimated correlation between social intelligence and reading and writing ability (*r* = 0.78) appears much higher than the correlation between emotional intelligence and reading and writing ability (*r* = 0.32; *p =* 0.04), the former estimate was based on only one identified effect size. Additional research correlating reading and writing ability with social reasoning is likely to modify that value. 

These more specific comparisons laid the groundwork for testing Hypothesis 3.

### 13.3. Personal and Emotional Intelligences Exhibit a Greater Difference (i.e., Lower Correlation) with Thing-Centered Intelligences than Does Social Intelligence (Hypothesis 3)

Our third and final hypothesis was that emotional and personal intelligences, when compared with social intelligences, would be relatively distinct from thing-centered intelligences, and that social intelligence would be most highly related to the mixed- and thing-centered abilities. As shown towards the bottom of Table 5, this was not exactly the case. Looking at the thing-centered rows, where one might expect the greatest difference, the correlations for social intelligence ranged from *r* = 0.22 to 0.30; those for emotional intelligence, from *r* = 0.17 to 0.29; and for personal intelligence, from *r* = 0.18 to 0.26. The differences seemed fairly small and did not reach statistical significance, excepting a marginally significant difference in the estimated relations of social and emotional intelligences with visuospatial processing (*r* = 0.29 versus *r* = 0.17, respectively, *p* = 0.06). Of note also, however, is that social intelligence was quite distinct from emotional intelligence, correlating at *r* = 0.23––no higher than with thing intelligences. Measures of social intelligence also correlated with speed and short-term memory, whereas the relation was near-absent for personal and emotional intelligences.

## 14. Exploratory Factor Analyses

In further analyses, we constructed a matrix of the 11 intelligences identified in our work by drawing on the estimated correlations reported in Table 5 (the full matrix can be found in Supplemental Table 5, in the “Exploratory Factor Analysis” section of (Bryan and Mayer 2021a)). Missing correlations for a given intelligence were imputed by taking the average of its correlations with the other intelligences in the table. Note that each correlation in the matrix stemmed from a different number of studies, with different *Ns*. We used both SPSS and R to conduct exploratory factor (and principal components) analyses. Unsurprisingly, perhaps, our attempts to factor analyze the matrix sometimes identified the matrix as singular, and also generated frequent ultra-Heywood cases (factor loadings above 1.00) regardless of the software. 

To ameliorate the issue, we focused on a smaller matrix of six intelligences that were especially relevant to our hypotheses: three people-centered intelligences and three mixed and thing-centered intelligences (see Table 5). We excluded fluid intelligence and comprehension knowledge because their breadth tends to promote Heywood cases in factor loadings (Bryan and Mayer 2020) and excluded processing speed and short-term memory because as process-based or ‘utility’ intelligences, they were less-relevant to the people-thing continuum (see the Introduction section).

Using the six-by-six matrix, we were able to obtain a solution that extracted three principal components in R shown in Table 6 (left) (a solution in SPSS using a slightly modified matrix was nearly identical). We also report a Schmid–Leiman factor transformation in Table 6 (right). The Schmid–Leiman allows for a hierarchical factor analysis that includes a first general factor in the context of exploratory factor analysis (Most hierarchical factor solutions today are in the context of confirmatory factor analysis). The root mean square of the residuals (RMSR) was 0.09 for the principal components solution and 0.05 for the Schmid–Leiman; the latter, however, generated an ultra-Heywood case that was then reduced by the software in the solution indicated.

Encouragingly, however, both the principal components and Schmid–Leiman share key aspects in common. They begin with a *g* or *g*-like intelligence factor. Beyond that, the principal components analysis next extracted a clear bipolar people versus thing-centered dimension of broad intelligences, with visuospatial processing and quantitative knowledge loading negatively and emotional and personal intelligences positively. The third factor was defined by a social–verbal composite at one end (social and reading and writing intelligences), versus visuospatial processing at the other. The Schmid–Lehman solution similarly contrasted personal and emotional intelligences on their own second factor (after *g*), separating them from a third, visuospatial and quantitative knowledge factor. The first factor after *g* was the reading and writing ability and social intelligence combination that appeared third in the principal components analysis. These results are consistent with our prediction of a distinct subgroups of mental abilities, akin to a people–thing continuum. A viable alternative interpretation is possible, however: that emotional and personal intelligences at least might form a single broad intelligence. As more studies accumulate, the distinction (or lack thereof) ought to become clearer.

## 15. Publication Bias

**Figure 4:**
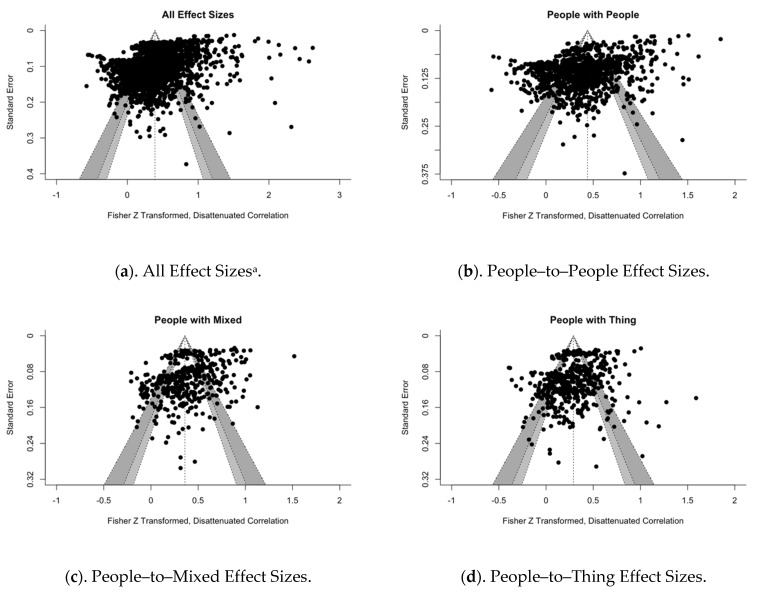
Funnel plots depicting the disattenuated, transformed effect sizes (**a**) for all studies in our sample and those (**b**) people-to-people, (**c**) people-to-mixed, and (**d**) people-to-thing comparisons. The white, light gray, and dark gray shading on each funnel plot represent the 90, 95, and 99% pseudo confidence intervals. The small, dotted vertical line represents the unweighted, average effect sizes. ^a^ Includes mixed-to-mixed, mixed-to-thing, and thing-to-thing correlations.

Lastly, we tested for the presence of publication bias among our sample of studies. Because it was unclear what kind of bias might exist in this heterogeneous group of studies, we created four funnel plots each comparing the Fisher’s Z-transformed, disattenuated correlations against their respective standard errors. The first funnel plot represented all the effect sizes, while the remaining three plotted effect sizes that belonged to people-to-people, people-to-mixed, and people-to-thing pairs, given the importance of these to our central hypotheses (see Figure 4). The effect sizes clustered in the middle and towards the apex of the funnel in all four plots.

There existed noticeable spread across the upper portions of each plot, suggesting that studies with larger samples reported greater variability in effect sizes, especially for people-to-people-centered abilities (see Figure 4b). Sample size was a significant, positive moderator of effect size among the people-to-people and people-to-mixed although not so for people-to-thing contrasts, suggesting that larger correlations were reported by studies with larger sample sizes (*p’s* < 0.01) (Hox and De Leeuw 2003). Effect sizes associated with studies using smaller sample sizes were less common but demonstrated some small spread across the lower portions of each plot. Funnel plot asymmetry depicting such large-study effects has been noted in the intelligence literature, although bias in the field generally trends in the opposite direction, indicating small-study effects (Nuijten et al. 2020).

To our knowledge, little research has focused on understanding the factors that contribute to large-study effects in funnel plots. Some possibilities might include distortions stemming from corrected correlations of scales with low initial reliabilities, or certain people-centered measures relative to others. More detailed examinations of the dispersion of effect sizes for specific intelligence contrasts were inconclusive (see “Publication Bias” in the Technical Supplement; Bryan and Mayer 2021a). Aside from that, the substantial clustering of effect sizes, particularly in the plots depicting all effect sizes and the people-to-people correlations, may be due to chance, or an artifact of the presence of additional moderators beyond the scope of our focus here (Sterne et al. 2005).

## 16. Discussion

A century of intelligence research followed upon Thorndike’s (1920) proposed social intelligence up to our present-day understanding of people-centered intelligences (Mayer 2018). Despite the hundred years of research since, remarkably little direct evidence has addressed whether people-centered intelligences are psychometrically distinct from other broad intelligences such as comprehension knowledge, visuospatial processing, or quantitative knowledge, until recently.

The present research reports a key, direct test of the distinction between people-centered and more traditional thing-focused mental abilities. Our aim was to establish an understanding of the average relation between different types of mental abilities, classified according to the problem-solving area of each—i.e., people-centered, thing-centered, or mixed. We proposed that people-centered abilities, which people draw upon to reason about themselves and others, relate more highly with one another than with mental abilities about things (e.g., numbers and spatial relations). Additionally, we sought to explore whether the relations formed a relatedness-gradient (i.e., through mixed intelligences) consistent with a people-versus-thing continuum.

## 17. Are People-Centered Intelligences Distinct from Other Abilities?

Across 87 studies including more than 2000 effect sizes, our findings provide evidence supporting distinct correlational differences between people- and thing-centered intelligences. Specifically, people-centered intelligences were plainly more highly correlated among themselves than they were with thing-centered intelligences at *r* = 0.43 compared to *r* = 0.29 (and relations with mixed intelligences were in-between at *r* = 0.36). This pattern was robust when examining each people-centered ability and their respective relations to different types of thing-centered and mixed abilities. In a parallel fashion, thing-centered intelligences were correlated more highly with one another than they were with people-centered intelligences, at *r* = 0.74, versus 0.29. Together, these findings provide key evidence supporting the proposed distinction between classes of people- and thing-centered intelligences, with mixed-centered abilities in between—a people–thing continuum that arguably also showed up in a provisional factor analysis of a composite matrix.

## 18. An Observation on the “Cohesiveness” of the Intelligence Groups

Alongside the evidence for the continuum above, there were differences in the magnitude of the average correlations of the people-to-people intelligences versus thing-to-thing and mixed-to-mixed intelligences (*r* = 0.43 compared to 0.74 and 0.62, respectively). Most obviously, this appears due to the relatively lower correlations that existed between measures of social intelligence with personal and emotional intelligence (e.g., *r* = 0.23 with emotional intelligence). Indeed, the exploratory factor analyses indicated that social intelligence was closer to Grw than to its neighboring people-centered measures. A further distinction between social and other people-centered intelligences was that measures of social intelligence correlated with speed and short-term memory, whereas the relation was near-absent for personal and emotional intelligences. It may be that, compared to assessments of social intelligence, the tests used to assess personal and emotional intelligences represented here tap into more domain-specific knowledge than basic processing abilities. Additional research may further elucidate the relations between other people-centered abilities and more low-level processing capacities (Fiori et al. 2019).

Lee J. Cronbach had pointed out 60 years ago that social intelligence was challenging to distinguish from general reasoning (Conzelmann et al. 2013; Cronbach 1960). Some experts have remarked that differences in social reasoning skills may be more heavily tied to an individual’s verbal and abstract reasoning skills than initially supposed (Kihlstrom and Cantor 2000; R. L. Thorndike and Stein 1937). Here, it appeared not-so-highly related to *any* other intelligence (except, in one study, to reading-writing ability). The findings here are obviously discrepant with Cronbach’s concern that social intelligence merged into general intelligence. If these results are taken at face value, social intelligence appears to correlate with little else; perhaps, however, as more reliable and better-defined assessments of the social intelligence are developed they may prove more highly related to other people-centered intelligences (Conzelmann et al. 2013; Lee et al. 2000; Walker and Foley 1973).

## 19. Strengths and Limitations

The current meta-analysis complements previous research examining the relations among people-centered intelligences and more traditionally studied mental abilities such as fluid intelligence and comprehension knowledge (Olderbak et al. 2018; Schlegel et al. 2019b; Völker 2020). Indeed, our estimates for the correlations between emotional intelligence and fluid intelligence (*r* = 0.29) and emotional intelligence with comprehension knowledge (*r* = 0.35) are well in range of the values found by Olderbak et al. (2018), who reported estimates for the branches of emotional intelligence with fluid intelligence ranging from 0.21 to 0.50 (total EI *r* = 0.33), and with comprehension knowledge ranging from 0.18 to 0.39 (total EI *r* = 0.26). Our findings also complement this body of literature by providing some of the first estimates for how other people-centered intelligences, such as social and personal intelligence, correlate with other mental abilities.

That said, our study exhibits some important limitations. Our literature search focused on identifying relevant works that correlated people-centered intelligences with thing-centered and mixed abilities. As such, the average thing-to-thing, thing-to-mixed, and mixed-to-mixed correlations were estimated only from those studies in our sample that provided such correlations rather than the broader intelligence literature. Nonetheless, the average correlations among mixed and thing-centered abilities found here were comparable to estimates reported in both meta-analyses and large-scale psychometric studies in the field (e.g., Bryan and Mayer 2020; Phelps et al. 2005; Sanders et al. 2007). For example, Bryan and Mayer reported correlations among thing-centered abilities, including fluid intelligence, visuospatial processing, and quantitative knowledge, averaging *r* = 0.69 (range *r* = 0.58 to 0.81)—approximating the average thing-to-thing centered estimate of *r* = 0.74 (range *r* = 0.68 to 0.77) found in this report. (Estimates taken from Bryan and Mayer were produced from factor modeling, which are considered corrected for unreliability and are, therefore, approximately comparable to the findings here.) Additionally, the estimates for correlations between thing-centered and mixed intelligences reported by Bryan and Mayer (2020) averaged *r* = 0.53 (range *r* = 0.42 to 0.73), whereas our values here overlapped, albeit they were somewhat lower, averaging *r* = 0.43 (range 0.34 to 0.62).

A further limitation of our findings includes possible alternative interpretations of what we regard as a people–thing continuum among select broad intelligences. Although our factor analyses support such a dimension, it does not rule out the possibility that two or three of the emotional, personal, and social intelligences may constitute a single “broader” people-centered reasoning capacity with subfacets of personal, emotional, and (possibly) social intelligences. Additionally, our middle, “mixed” category of intelligences might exhibit its “betweenness” because verbal ability is required to some degree to understand and answer both the people- and thing-centered measures.

Finally, the persistent criticisms of people-centered intelligences—and especially social intelligence—through the mid-to-late 20th century tamped down research in the area (e.g., Walker and Foley 1973), with notable exceptions (e.g., Guilford 1988). Researchers shied away from the construct for some decades thereafter, although interest was reignited by Gardner’s (1983) biopsychological conception of multiple intelligences. Such interest was further piqued by the advent of emotional intelligence in the early 1990s (Mayer et al. 1990; Salovey and Mayer 1990), and more recent conceptualizations of emotion recognition ability and social intelligence (Conzelmann et al. 2013; Kihlstrom and Cantor 2000; Lee et al. 2000; Schlegel et al. 2019b). Personal intelligence, in particular, was introduced so recently that its comparisons with the other scales are limited to date. Our empirical tests were more constrained in scope than we might have liked given these realities, but we are hopeful further research will remediate these issues moving forward.

## 20. Conclusions

There is converging evidence as to the importance of people-centered intelligences in real life. Such findings include that people-centered intelligences out-predict thing-centered intelligences in regard to certain life outcomes, especially in relation to work and school settings that require reasoning about people (Mayer et al. 2018; Mayer and Skimmyhorn 2017). In addition, interventions designed to improve interpersonal understanding positively affects behavior (Durlak et al. 2011; Taylor et al. 2017). When these findings are combined with the present analyses, they collectively argue for the existence of a partially distinct group of intelligences that concern reasoning about people.

Because we have found evidence in support of such distinctions, it is worth considering the new reality of enhanced research activity in regard to these broad intelligences. Continued research in the area is warranted, focusing on the relations among people- and thing-centered broad intelligences, as well as continuing investigations as to what such people-centered intelligences uniquely predict. Such work can consolidate our understanding of how this diverse set of abilities best fit among other, more traditionally studied intelligences, as well as their practical importance to individuals and society. 

## Figures and Tables

**Table 1:** Index of key assessments for people-centered abilities.

**Emotional Intelligence**	
***Broad Scales ^a^***	
*Omnibus measures of multiple areas of emotional intelligence.*	▪ Mayer–Salovey–Caruso Emotional Intelligence Scale (MSCEIT; Mayer et al. 2003). ▪ Multifactor Emotional Intelligence Scale (MEIS; Mayer et al. 1999). ▪ Test of Emotional Intelligence (TEMINT; Blickle et al. 2011). ▪ Geneva Emotional Competence Test (GECo; Schlegel and Mortillaro 2019). ▪ Situational Judgement Test of Emotional Intelligence (SJIT; Sharma et al. 2013).
***Specific Scales***	
***Emotion Recognition Ability***	
*Measures of specific ability to accurately identify emotions in oneself and others. Includes perceiving emotions across expression modalities, including faces, voices, and the body.*	▪ Geneva Emotion Recognition Test (GERT and GERT-S; Schlegel et al. 2014; Schlegel and Scherer 2016). ▪ Multimodal Emotion Recognition Test (MERT; Bänziger et al. 2009). ▪ Emotion Recognition Index (ERI; Scherer and Scherer 2011). ▪ Japanese and Caucasian Brief Affect Recognition Test (JACBART; Matsumoto et al. 2000). ▪ Profile of Nonverbal Sensitivity (PONS and MiniPONS; (Bänziger et al. 2011; DePaulo and Rosenthal 1979). ▪ Diagnostic Analysis of Nonverbal Accuracy (DANVA; Pitterman and Nowicki 2004). ▪ Reading the Mind in the Eyes Test (RMET; Baron-Cohen et al. 2001). ▪ Index of Vocal Emotion Recognition (Vocal-I; Scherer et al. 2001). *Moreover, relevant subscales of the MSCEIT*
***Emotion Understanding and Management***	
*Assessments of understanding how situations or events are linked to emotional experiences and, for management, of effective regulation of emotions in the self and others. Involves strategies aimed at maintaining or enhancing positive emotional experiences and reducing/regulating negative ones.*	▪ Geneva Emotional Knowledge Test (GEMOK; Schlegel and Scherer 2018). ▪ Multimedia Emotion Management Assessment (MEMA; MacCann et al. 2016). ▪ Situational Test of Emotional Understanding (MacCann and Roberts 2008). ▪ Situational Test of Emotional Management (MacCann and Roberts 2008). *Moreover, relevant subscales of the MSCEIT, MEIS, and GECO*
**Social Intelligence**	
***Broad Scales ^b^***	
*Omnibus measures of multiple areas of social intelligence.*	▪ George Washington Social Intelligence Test (GWSIT; Hunt 1928). ▪ Magdeburg Test of Social Intelligence (MTSI; Conzelmann et al. 2013). ▪ Four Factor Test (O’Sullivan and Guilford 1975).
***Social Perception***	
*Measures of the capacity to understand behavioral expressions that convey people’s attitudes, or underlying intentions, and feelings. Modalities include facial expression, hand gestures, posture, and vocalizations.*	▪ Interpersonal Perception Task (IPT; Costanzo and Archer 1989). *Moreover, relevant subscales of the GWSIT, Four Factor Test, and MTSI*
***Social Knowledge***	
*Tests for knowledge of social etiquette and rules. Largely tied to environmental or cultural factors*.	▪ Tacit Knowledge Inventory (Wagner and Sternberg 1987).
***Social Insight, Memory and Understanding***	
*Assessments of the capacity to reason about behavioral sequences, including the antecedents of behavior and the resulting consequences of one’s behavioral choices. Involves understanding social cues and choosing behaviors that lead to desired social outcomes.*	▪ Chapin Social Insight Test (Gough 1965). *Moreover, relevant subscales of the GWSIT and Four Factor Test*
**Personal Intelligence**	
***Broad Scales***	
*Measures of the capacity to understand personality in oneself and others.*	▪ Test of Personal Intelligence (TOPI and TOPI-MINI; Mayer et al. 2012, 2019).

^a^ Many of the broad scales of emotional intelligence also provide subscale scores for measuring specific areas of emotional intelligence and sometimes appeared in the research corpus studied in this meta-analysis. ^b^ As above, many of the broad scales of social intelligence also provide subscale scores for measuring specific areas of social intelligence and sometimes appeared in the research corpus studied in this meta-analysis.

**Table 2:** List of included studies.

Article		N	Mental Ability Represented and Assessment(s)					
			Person-Centered		Mixed		Thing-Centered	
			Mental Ability	Assessments	Mental Ability	Assessments	Mental Ability	Assessments
Austin (2004)		92	Gei	Ekman-60	Grw	National Adult Reading Test		
Austin (2005)		95	Gei	Ekman-60			Gf	Raven’s Matrices
Austin (2010)		135	Gei	MSCEIT; STEU; STEM	Gc	Quickie Battery Vocabulary	Gf	Quickie Battery Letter Series
Barchard (2003)		150	Gei	MSCEIT	Gc	French Kit		
			Gsi	Four Factor Test				
Bastian et al. (2005)		246	Gei	MSCEIT	Glr	PWAT	Gf	Raven’s Matrices
Brackett and Mayer (2003)		207	Gei	MSCEIT	Gc/Grw	Verbal SAT		
Brackett et al. (2006)		316	Gei	MSCEIT	Gc/Grw	Verbal SAT		
Broom (1930)		646	Gsi	GWSIT	Grw	Thorndike Reading Comprehension		
Campbell and McCord (1996)		50	Gsi	Chapin Social Insight Test	Gc	WAIS—R Comprehension	Gv	WAIS—R Pic. Arrangement
Checa and Fernández-Berrocal (2015)		92	Gei	MSCEIT	Gc	KBIT Vocabulary	Gf	KBIT Matrices
Conzelmann et al. (2013)								
	Study 1	127	Gsi	Magdeburg Test	Gc	BIS Verbal	Gv	BIS Figural
							Gsm	BIS Memory
							Gs	BIS Speed
	Study 2	190	Gsi	Magdeburg Test	Gc	BIS Verbal	Gv	BIS Figural
							Gsm	BIS Memory
							Gs	BIS Speed
Cook and Saucier (2010)		88	Gei	Eyes Test			Gv	Mental Rotation Test
Côté and Miners (2006)		175	Gei	MSCEIT			Gf	Culture Fair Test
Coyle et al. (2018)		249	Gei	Eyes Test	Gc	ACT English; Reading	Gq	ACT Math
Curci et al. (2013)		183	Gei	MSCEIT	Gc	WAIS Vocabulary	Gf	Raven’s Matrices
Dacre Pool and Qualter (2012)		1086	Gei	MSCEIT			Gf	Raven’s Matrices
Di Fabio and Palazzeschi (2009)		124	Gei	MSCEIT			Gf	Raven’s Matrices
Di Fabio and Saklofske (2014)		194	Gei	MSCEIT			Gf	Raven’s Matrices
Evans et al. (2020)		830	Gei	STEU; STEM; Eyes Test	Gc	ICAR Verbal	Gf	ICAR Letter and Number Series
Farrelly and Austin (2007)								
	Study 1	99	Gei	MSCEIT	Gc	Quickie Battery Vocab/Analogies	Gf	Quickie Battery Letter Series/ Matrices
	Study 2	199	Gei	MSCEIT	Gc	Quickie Battery Vocab/Analogies	Gf	Raven’s Matrices
Fiori and Antonakis (2011)		149	Gei	MSCEIT			Gf	Culture Fair Test
Fiori and Antonakis (2012)		85	Gei	MSCEIT			Gf	Culture Fair Test
Habota et al. (2015)		69	Gei	Ekman-60; Eyes Test	Glr	Rey Auditory Verbal Learning		
Holmes et al. (1976)		45	Gsi	Chapin Social Insight Test			Gf	Shipley Abstract Reasoning
Ivcevic et al. (2007)								
	Study 1	107	Gei	MSCEIT	Glr	Remote Associates Test		
	Study 2	113	Gei	MSCEIT	Gc/Grw	SAT Verbal	Gq	SAT Math
					Glr	Remote Associates Test		
Karim and Weisz (2010)		192	Gei	MSCEIT			Gf	Raven’s Matrices
Keating (1978)		117	Gsi	Chapin Social Insight Test	Gc		Gf	Raven’s Matrices
Kokkinakis et al. (2017)		56	Gei	Eyes Test			Gf	WASI-II Matrix
Lanciano and Curci (2014)		89	Gei	MSCEIT			Gf	Raven’s Matrices
Lee et al. (2000)		169	Gsi	GWSIT; Four Factor Test	Gc	WAIS-R Vocabulary; Verbal Analogies	Gf	Spatial Analogies; WAIS-R Pic. Completion
Libbrecht and Lievens (2012)		764	Gei	STEU; STEM			Gf	Flemish Gf test
Lopes et al. (2006)		44	Gei	MSCEIT	Gc	Mill Hill Vocabulary		
Lopes et al. (2003)		103	Gei	MSCEIT	Gc	WAIS-III Vocabulary		
Lopes et al. (2005)		76	Gei	MSCEIT	Gc	Mill Hill Vocabulary	Gf	Culture Fair Test
					Gc/Grw	SAT Verbal	Gq	SAT Math
Lumley et al. (2005)		140	Gei	MSCEIT	Grw	Wide Range Achievement Test		
MacCann et al. (2014)		688	Gei	MSCEIT	Gc	French Kit Vocab; ETS Analogies and Sentence Completion	Gf	French Kit Letter Sets, Figure Class. and Calendar
					Glr	French Kit Word Endings, Word Beginnings, and Opposites.	Gv	French Kit Cube Comp., Hidden Patterns, Surface Development
							Gq	French Kit Math Aptitude, Necessary Math., Subtraction and Multiplication.
MacCann et al. (2016)		394	Gei	MSCEIT	Gc	French Kit Vocab, Analogies, Sentences	Gf	French Kit Letters, Figures, Calendar
MacCann et al. (2011)		118	Gei	STEU; STEM	Gc	IST Knowledge	Gf	Raven’s Matrices
					Grw	ACER—Reading Comprehension		
MacCann and Roberts (2008)		200	Gei	STEU; STEM; MEIS Stories	Gc	Gf/Gc Quickie Battery Vocabulary		
Martin and Thomas (2011)		87	Gei	MSCEIT			Gf	Raven’s Matrices
Mayer et al. (1999)		500	Gei	MEIS	Gc	Army Alpha Vocabulary		
Mayer et al. (2018)								
	Study 1	394	Gpi	TOPI MINI	Gc	Wordsumplus; Modified Vocabulary	Gf	Backwards digit span
	Study 2	492	Gpi	TOPI 1.4	Gc	Wordsumplus		
Mayer et al. (2012)								
	Study 1	241	Gpi	TOPI 1.0	Gc	Modified Vocabulary		
	Study 2	308	Gpi	TOPI 1.1	Gc	Modified Vocabulary		
	Study 3	385	Gpi	TOPI 1.2	Gc	Modified Vocabulary		
			Gei	MSCEIT; Eyes Test				
Mayer and Skimmyhorn (2017)								
	Study 1	932	Gpi	TOPI	Gc/Grw	SAT Verbal	Gq	SAT Math
							Gv	O*Net Spatial Ability
	Study 2	893	Gpi	TOPI	Gc/Grw	SAT Verbal	Gq	SAT Math
							Gv	O*Net Spatial Ability
McIntyre (2010)		420	Gei	MSCEIT	Gc	French Kit Vocabulary		
Miller and Lenzenweger (2012)		93	Gei	PONS			Gv	Digit Symbol Coding
Nowicki and Duke (1994)		1144	Gei	DANVA	Gc	CTBS—Vocab; Word Recognition	Gq	CTBS—Math Concepts; Comprehension; Counting
					Grw	CTBS—Reading Comp.; Spelling		
O’Sullivan and Guilford (1975)		240	Gsi	Four Factor Test	Gc	Henmon-Nelson Vocab; Verbal Analogies, Classification, Comprehension	Gf	DAT Abstract Reasoning; Figure Matrix
							Gq	ITED Quantitative Thinking
Olderbak et al. (2015)								
	Study 1	484	Gei	DANVA; Eyes Test	Gc	ETS Vocabulary		
	Study 2	210	Gei	DANVA; Eyes Test	Gc	ETS Vocabulary		
Peters et al. (2009)		50	Gei	MSCEIT-YV	Grw	WJ-III Reading; SAT Reading	Gq	WJ-III Math; SAT Math
Peterson and Miller (2012)		45	Gei	Eyes Test	Gc	WASI Vocabulary	Gf	WASI Matrix Reasoning
Pickett et al. (2004)		46	Gei	DANVA			Gq	ETS-Quantitative
Riggio et al. (1991)		171	Gsi	Four Factor Test	Gc	WAIS-R Vocabulary; Shipley Vocabulary	Gf	Shipley Abstract Reasoning
Roberts et al. (2006)		138	Gei	MSCEIT	Gc	Quickie Battery Vocabulary, Esoteric Analogies	Gf	Matrices, Swaps
Rosete and Ciarrochi (2005)		41	Gei	MSCEIT	Gc	WASI Verbal	Gf	WASI Performance
Schellenberg (2011)		106	Gei	MSCEIT	Gc	KBIT Verbal	Gf	KBIT Performance
Schlegel and Mortillaro (2019)								
	Study 1	149	Gei	ERI; STEU; STEM; GECo			Gf	NV5-R Inductive Reasoning
	Study 2	187	Gei	MSCEIT; STEU; STEM; GECo			Gf	Culture Fair Test
	Study 4	206	Gei	GECo	Gc	IST Verbal	Gv	IST Figural
							Gq	IST Numeric
Schlegel and Scherer (2016)		128	Gei	GERT; STEU; STEM			Gf	Culture Fair Test
Schlegel and Scherer (2018)								
	Study 1	159	Gei	GERT; DANVA; ERI; GEMOK	Gc	Shipley Vocabulary		
	Study 4	103	Gei	GERT; DANVA; ERI; GEMOK			Gf	Culture Fair Test
Schlegel et al. (2019a)		131	Gei	GERT; MERT; MiniPONS; JACBART; MSCEIT	Gc	NV5-R Vocabulary	Gf	NV5-R Reasoning
Schlegel et al. (2017)		214	Gei	GERT			Gf	Culture Fair Test
Sharma et al. (2013)		147	Gei	SJT-EI	Gc	Mill Hill Vocabulary	Gf	Raven’s Matrices
Śmieja et al. (2014)		4624	Gei	TIE	Gc	Cattell-Horn Word Classification	Gf	Raven’s Matrices
Sternberg and Smith (1985)		101	Gsi	Chapin Social Insight Test; GWSIT			Gf	Culture Fair Test
							Gv	Embedded Figure Test
Thorndike and Stein (1937)		500	Gsi	GWSIT	Gc	Thorndike Vocabulary	Gq	Thorndike Arithmetic Reasoning
					Grw	Thorndike Comprehension		
Völker (2020)		188	Gei	GECo	Gc	INSBAT General Knowledge; Verbal Fluency; Word Meaning	Gf	INSBAT Inductive; Verbal Deductive
							Gv	INSBAT Figural
Warwick and Nettelbeck (2004)		84	Gei	MSCEIT			Gf	DAT Abstract Reasoning
Webb et al. (2014)		65	Gei	MSCEIT	Gc	WASI Verbal	Gf	WASI Performance
Weis and Süß (2007)		101	Gsi	Magdeburg Test			Gf	BIS Reasoning
							Gsm	BIS Memory
							Gs	BIS Speed
Wickline et al. (2012)		42	Gei	DANVA			Gv	WISC-III Picture Arrangement
Wong et al. (1995)								
	Study 1	143	Gsi	GWSIT; Four Factor Test	Gc	WAIS-R Vocabulary	Gv	WAIS-R Pic. Completion
	Study 2	240	Gsi	GWSIT; Four Factor Test	Gc	Verbal Analogies	Gv	Spatial Analogies

Note. Gc = comprehension knowledge; Gei = emotional intelligence; Gf = fluid intelligence; Glr = long-term retrieval; Gpi = personal intelligence; Grw = reading and writing ability; Gs = processing speed; Gsi = social intelligence; Gsm = short-term memory; Gv = visuospatial processing; Gq = quantitative knowledge.

**Table 3:** Characteristics of included studies.

Sample Type ^a^		
	University	58 studies
	Community	13 studies
	Online	7 studies
	Child/adolescent	7 studies
	Clinical	4 studies
	Other	4 studies
Sample size		mean = 283.20, total = 24,638; range = 41 to 4642
Gender (males to females)		56% female; males = 9773; females = 12,439
Age of participants		mean = 25.52; range = 13.3 to 69.8
Publication year		mean = 2007; median = 2010; range = 1930 to 2020
Reliability ^b^		
	Social intelligence	mean = 0.63; range = 0.10 to 0.98.
	Emotional intelligence	mean = 0.74; range = 0.42 to 0.99
	Emotion recognition ability	mean = 0.73; range = 0.43 to 0.95
	Personal intelligence	mean = 0.87, range = 0.71 to 0.94

^a^ Some studies recruited participants of more than one type and so the total exceeds 87 (e.g., participants were recruited from the community and university). ^b^ Average reliability for social, emotional, and personal intelligences included instances where the reliability was estimated from other sources (see Estimating Reliabilities, above).

**Table 4:** Associated statistics for the estimated average correlation among intelligence comparison types.

Contrast	*k*	*N*	*Avg. Reliability*	*r_est._*	95% CI
People–with–People	1085	15,893	0.68	0.43	[0.39, 0.48]
People–with–Mixed	424	16,953	0.72	0.36	[0.31, 0.40]
People–with–Thing	464	13,751	0.73	0.29	[0.24, 0.34]
Thing–with–Mixed	117	6630	0.78	0.43	[0.37, 0.49]
Mixed–with–Mixed	66	3329	0.72	0.62	[0.57, 0.67]
Thing–with–Thing	58	3463	0.76	0.74	[0.70, 0.78]

**Table 5:** Average estimated correlations ^a^ among people-centered, mixed, and thing-centered intelligences organized by type of people-centered ability.

		Social Intelligence				Emotional Intelligence ^b,c^				Personal Intelligence			
Class and Subclass of Intelligence		*k*	*N*	*r*	95% CI	*k*	*N*	*r*	95% CI	*k*	*N*	*r*	95% CI
*People-centered intelligences*													
	Social intelligence (Gsi)	621	1894	0.33	[0.28, 0.38]	21	468	0.23	[0.07, 0.37]	--	--	--	--
	Emotional intelligence (Gei) ^b,c^	21	468	0.23	[0.07, 0.37]	440	13693	0.50	[0.45, 0.54]	3	352	0.70	[0.40, 0.87]
	Personal intelligence (Gpi)	--	--	--	--	3	352	0.70	[0.40, 0.87]	--	--	--	--
*Mixed intelligences*													
	Comprehension knowledge (Gc)	169	2209	0.38	[0.32, 0.44]	173	9015	0.35	[0.29, 0.41]	6	3218	0.41	[0.14, 0.62]
	Long-term retrieval (Glr)	8	225	0.10	[−0.13, 0.32]	32	1307	0.14	[0.02, 0.25]	--	--	--	--
	Reading and writing ability (Grw)	1	646	0.78	[0.35, 0.94]	42	2453	0.32	[0.22, 0.42]	2	1825	0.35	[−0.06, 0.66]
*Thing-centered intelligences*													
	Fluid intelligence (Gf)	98	1314	0.30	[0.23, 0.38]	168	9179	0.29	[0.22, 0.35]	--	--	--	--
	Visuospatial processing (Gv)	73	980	0.29	[0.21, 0.37]	31	1345	0.17	[0.05, 0.28]	2	2099	0.26	[−0.15, 0.60]
	Quantitative knowledge (Gq)	35	848	0.22	[0.11, 0.33]	63	2837	0.24	[0.14, 0.32]	2	1825	0.18	[−0.24, 0.54]
*Other mental abilities ^d^*													
	Processing speed (Gs)	41	391	0.29	[0.18, 0.39]	2	201	0.09	[−0.37, 0.51]	--	--	--	--
	Short-term memory (Gsm)	41	391	0.38	[0.28, 0.47]	4	164	-0.03	[−0.37, 0.32]	1	394	−0.02	[−0.56, 0.53]

^a^ All average correlation estimates are taken from the unweighted random effects models. Values are presented as Pearson r’s corrected for disattenuation due to reliability ^b^ The estimated correlations for emotional with social intelligence and emotional with personal intelligence have been duplicated in other columns. ^c^ Includes both measures labeled as emotional intelligence and emotion recognition ability. ^d^ The “other” abilities were regarded as process-based or “utility” intelligences and, although included here, were otherwise excluded from the people-to-thing intelligence analyses.

**Table 6:** Principal component loadings for the three-component unrotated solution testing the people–thing continuum.

Broad Intelligence	Principal Components Solution ^a^			Schmid–Lehman Analysis ^b^			
	I	II	III	*g*	I	II	III
***Thing-centered intelligences***							
Visuospatial processing	**0.60**	**−0.46**	**0.51**	**0.33**			**0.57**
Quantitative knowledge	**0.72**	**−0.52**	**0.30**	**0.56**			**0.84**
***Mixed intelligences***							
Reading and writing	**0.83**	−0.13	**−0.48**	**0.89**	**0.47**		
***People-centered intelligences***							
Emotional intelligence	**0.62**	**0.63**	0.26	**0.34**		**0.61**	
Personal intelligence	**0.66**	**0.61**	0.21	**0.41**		**0.92**	
Social intelligence	**0.71**	−0.06	**−0.60**	**0.67**	**0.37**		

Note. Factor loadings above 0.30 are bolded. ^a^ The principal components solution converged without warnings or issues. ^b^ The Schmid–Lehman was adjusted because it contained an ultra-Heywood case and the estimated weights may be somewhat incorrect.

## Appendix A. Designations of Assessments as Broad or Narrow

Akin to the broad and narrow abilities reflected in the Cattell–Horn–Carroll model, broader assessments included total scores on assessment that tapped into multiple areas of reasoning within a broad ability (e.g., MSCEIT total scores were calculated from participants’ scores on all four branches and eight tasks) while more narrow assessments were those that tapped into a single area of problem solving (e.g., we coded both the MSCEIT Faces task, and the Emotion Perception Branch as a whole (the Faces and Pictures tasks combined) both as narrow tasks relative to the total scale). Broader assessments were coded as “0” and narrow assessments were assigned a “1”. A table depicting the designations of each assessment identified in the current work is presented below (see Table A1).

**Table A1:** Categorization of broad and specific assessments, organized by intelligence type.

Intelligence Type and Measure		Categorization
***People-Centered Assessments***		
	MSCEIT	When MSCEIT total scores were reported, we considered the assessment to be broad. When branch or task scores were reported, the individual tasks were considered specific assessments of emotional reasoning.
	MEIS	When MEIS total scores were reported, we considered the assessment to be broad. When branch or task scores were reported, the individual tasks were considered specific assessments of emotional reasoning;
	STEU	Narrow; assesses a single area of emotion reasoning (Understanding).
	STEM	Narrow; assesses a single area of emotion reasoning (Management).
	TIE	Narrow; assesses four areas of emotion reasoning
	GECo	Narrow; assesses four areas of emotion reasoning.
	Chapin Social Insight	Narrow; assesses social insight, a specific skill underlying social intelligence
	GWSIT	Broad; includes a composite score of social intelligence. Subscales including Judgements in Social Situations, Recognition of Mental States, Observations, Memory for Names and Faces, and Sense of Humor were treated as specific assessments when scores were reported for each individually.
	Four Factor Tests	Narrow; all four major subscales were assessed individually and kept separate throughout analyses. Each of the four subscales also pertains to different areas of social reasoning (Cartoon Predictions and Missing Cartoons each pertain to social insight; Expressions Grouping pertains to social perception).
	Magdeburg Test	Narrow; all four subscales were assessed individually and kept separate throughout analyses. Each assesses different skills pertaining to social reasoning (i.e., social perception, social memory, social understanding/insight).
	RMET	Narrow; assesses ability to perceive emotions in the eyes.
	TOPI	Broad; assesses four areas of reasoning about personality.
	Tacit Knowledge Inventory	Narrow; all items assessing social etiquette/social knowledge.
	IPT-15	Narrow; assesses social perception drawing on 15 videos of social interactions.
	SJT-EI	Narrow; scores are reported four three areas of emotional reasoning: facilitating emotions, perceiving emotions, and understanding emotions. The items that comprised each area were homogenous.
	GERT	Narrow; assesses emotion recognition/perception.
	MERT	Narrow; assesses emotion recognition/perception.
	MEMA	Narrow; assesses emotion recognition/perception.
	ERI	Narrow; assesses emotion recognition/perception.
	DANVA	Narrow; assesses emotion recognition/perception.
	JACBART	Narrow; assesses emotion recognition/perception.
	Ekman-60	Narrow; assesses emotion recognition/perception.
	GEMOK	Narrow; assesses emotion knowledge.
	Vocal-I	Narrow; assesses emotion recognition/perception.
	Nim-Stim Faces	Narrow; assesses emotion recognition/perception.
	SEI-T	Narrow; assesses emotion recognition/perception.
	PONS and MiniPONS	Narrow; assesses emotion recognition/perception.
***Mixed Assessments***		
	Wordsumplus	Narrow; assesses vocabulary or lexical knowledge by having participants identify synonyms of words.
	Modified Vocab	Narrow; assesses vocabulary or lexical knowledge by having participants pick out the meaning of a given word.
	SAT Verbal	Broad; diverse set of items related to verbal reasoning and reading comprehension.
	Cattell–Horn Word Classification	Narrow; task assessing verbal reasoning.
	IST Verbal	Broad; authors calculated scores based on performance on three distinct subtests (sentence completion, analogies, and similarities).
	Phonetic Word Association Test	Narrow; assesses verbal fluency.
	Quickie Battery Vocab	Narrow; assesses vocabulary or lexical knowledge.
	ACT Reading	Broad; diverse set of items related to reading comprehension. Similar to SAT Verbal.
	ACT English	Broad; diverse set of items related to verbal reasoning. Similar to SAT Verbal.
	Thorndike Intelligence Examination Vocabulary	Narrow; assesses vocabulary or lexical knowledge.
	Thorndike Intelligence Examination Comprehension	Narrow; assesses reading comprehension.
	BIS Verbal	Broad; scores calculated from multiple subtests assessing different types of verbal reasoning.
	KBIT Verbal Composite	Broad; scores calculated based on performance on two subscales.
	Quickie Battery Analogies	Narrow; assesses verbal knowledge.
	French Kit Vocab	Narrow; assesses vocabulary or lexical knowledge.
	French Kit Word Endings	Narrow; assesses lexical speed/fluency.
	French Kit Word Beginnings	Narrow; assesses lexical speed/fluency.
	French Kit Opposites	Narrow; assesses lexical speed/fluency.
	ETS Analogies	Narrow; assesses verbal knowledge.
	ETS Sentence Completion	Narrow; assesses verbal knowledge.
	Henmon–Nelson Vocab	Narrow; assesses vocabulary or lexical knowledge.
	WAIS Vocab	Narrow; assesses vocabulary or lexical knowledge.
	ICAR Verbal Reasoning	Broad; includes items related to vocabulary, logic, and general knowledge.
	Co-operative Reading Comp Test	Narrow; participants only given a subset of items from this assessment.
	IST Knowledge	Narrow; participants only given a subset of items from this assessment.
	ACER Vocab	Broad; information ascertained about the assessment suggests it is similar to the SAT.
	Mill Hill Vocab	Narrow; assesses vocabulary or lexical knowledge.
	WJ Broad Reading	Broad; score was calculated based on performance on three subtests (calculation, math fluency, and applied problems).
	Quickie Vocab/Analogies Composite	Broad; composite of performance on vocab and analogies subtests from Quickie Battery. See Farrelly and Austin (2007).
*Thing-Centered Assessments*		
	Backward digit span	Narrow; task assessing working memory capacity.
	Gf Test	Broad; 50 diverse items including numeric, verbal, and figural narrows assessing fluid intelligence. See Libbrecht and Lievens (2012) for description.
	SAT Math	Broad; diverse items pertaining to math comprehension, mathematical reasoning, and mathematical knowledge.
	O*Net Spatial Ability	Narrow; task assessing visualization/spatial reasoning.
	Raven’s Matrices	Narrow; assesses abstract/inductive reasoning.
	Culture Fair Test Scale 2	Broad; comprised of scales including matrix reasoning, classifications, sequences, and geometric reasoning.
	IST Numeric	Narrow; task related to number sequence completion.
	IST Figural	Narrow; task involving matrix reasoning.
	Quickie Battery Letter Series	Narrow; task assessing abstract reasoning. One study by Farrelly and Austin (2007) combined Quickie letter series and matrices to form composite fluid reasoning scale. In that instance, the combination was treated as a *scale.*
	Quickie Battery Matrices	Narrow
	ACT Math	Broad; diverse items. Similar to SAT Math.
	Thorndike Intelligence Examination Arithmetical Reasoning	Narrow; measure assessing mathematical reasoning.
	Embedded Figures Test	Narrow; narrow task assessing field dependence/independence.
	BIS Figural	Broad; scores calculated from multiple subtests assessing different types of figural reasoning.
	BIS Numeric	Broad; scores calculated from multiple subtests assessing different types of numeric reasoning.
	BIS Reasoning	Broad; includes verbal, numeric, and figural reasoning.
	KBIT Performance	Narrow; single subtest (matrices) assessing abstract/inductive reasoning.
	Swaps	Narrow; task assessing abstract reasoning.
	French Kit Letter Series	Narrow; task assessing abstract reasoning.
	French Kit Figure Classification	Narrow; task assessing abstract reasoning.
	French Kit Calendar Test	Narrow; task assessing abstract reasoning.
	French Kit Math Aptitude	Narrow; task assessing mathematical knowledge.
	French Kit Math Operations	Narrow; task assessing mathematical knowledge.
	French Kit Subtraction/Multiplication	Narrow; task assessing mathematical reasoning.
	French Kit Cube Comparisons	Narrow; task assessing visual reasoning.
	French Kit Hidden Patterns	Narrow; task assessing visual reasoning.
	French Kit Surface Development	Narrow; task assessing visual reasoning.
	DAT Abstract Reasoning	Narrow; single task assessing abstract/inductive reasoning.
	ITED Quantitative Thinking Test	Narrow; single task assessing quantitative reasoning.
	WAIS Picture Completion	Narrow; task assessing visual closure.
	ICAR Letter and Number Series	Narrow; assesses abstract/logical reasoning.
	WASI-II Matrix	Narrow; single task assessing abstract/inductive reasoning.
	Spatial Analogies	Narrow; single task assessing visual processing.
	WJ Math	Broad; scores calculated from performance on three subtests, including calculation, math fluency, and applied problems.
	WASI Performance	Broad; scores calculated from performance on block design and matrix reasoning tasks.
